# PARP Inhibitors in Breast Cancer: a Short Communication

**DOI:** 10.1007/s11912-023-01488-0

**Published:** 2024-01-02

**Authors:** Gordon R. Daly, Maen Monketh AlRawashdeh, Jason McGrath, Gavin P. Dowling, Luke Cox, Sindhuja Naidoo, Damir Vareslija, Arnold D. K Hill, Leonie Young

**Affiliations:** 1 The Department of Surgery, Royal College of Surgeons in Ireland, Dublin, Ireland; 2 The Department of Surgery, Beaumont Hospital, Dublin, Ireland

**Keywords:** PARP inhibitor, Breast cancer, BRCA, Homologous repair deficiency

## Abstract

**Purpose of Review:**

In the last decade, poly (ADP-ribose) polymerase (PARP) inhibitors have been approved in the treatment of several cancers, such as breast and ovarian cancer. This article aims to discuss the current uses, limitations, and future directions for PARP inhibitors (PARPis) in the treatment of breast cancer.

**Recent Findings:**

Following the results of the OlympiAD and EMBRACA trials, PARPis were approved in HER2-negative breast cancer with a germline BRCA mutation. We reviewed this class of drugs’ mechanism of action, efficacy, and limitations, as well as further studies that discussed resistance, impaired homologous recombination repair (HRR), and the combination of PARPis with other drugs.

**Summary:**

Improving understanding of HRR, increasing the ability to target resistance, and combining PARPis with other novel agents are continuing to increase the clinical utility of PARPis.

## Introduction

Breast cancer is the most diagnosed malignancy worldwide, with around 2.26 million cases in 2020 [1]. It is estimated that 1 in every 8–10 women will get breast cancer in their lifetime [2]. Breast cancer can be categorized based on the presence or absence of specific molecular markers: hormone (progesterone or estrogen) receptor (HR) positive/human epidermal growth factor-2 (HER2)-negative (70% of patients); HER2-positive (15–20%); and lastly triple-negative breast cancers (~15%) [3]. The discovery of these molecular subtypes combined with earlier detection has dramatically improved survival; according to the American Cancer Society’s “Cancer Facts and Figures 2022,” the breast cancer death rate dropped 42% from its peak in 1989 compared to 2019 [4]. Triple-negative breast cancer (TNBC) tends to have the worst prognosis, with stage I tumors having a 5-year cancer-specific survival rate of 85% compared to 94% and 99% for HER2-positive and HR-positive breast cancers, respectively, and a stage IV median survival of 1 year for TNBC compared to a median of 5 years for HER2-positive and HR-positive tumors. In addition, molecular subtype identification has allowed for better-personalized therapy, with HR+ tumors receiving endocrine therapy +/− chemotherapy, HER2-positive tumors receiving HER2-directed antibody therapy+ chemotherapy, and TNBC receiving chemotherapy alone as standard [3].

In the last 20 years, the advent of genomics has allowed the analysis of tumors on a molecular level, informing treatment decisions based on specific gene mutation status. Up to 10% of breast cancers have an underlying germline DNA mutation, typically in genes necessary for DNA repair and cell cycle checkpoint activators [5]. The most well-studied mutations are those in the BRCA1 and BRCA2 genes. BRCA mutations impair homologous recombination repair (HRR), resulting in genomic instability. BRCA mutations follow an autosomal dominant inheritance, increasing breast and ovarian cancer susceptibility. Specifically, a 45 to 75% lifetime risk of breast cancer and an 18 to 40% lifetime risk of ovarian cancer. In addition, BRCA-related breast cancer tends to be triple-negative and high-grade, making it typically more aggressive than sporadic breast cancer [6]. HRR is a complex pathway, and the mutation of several other genes, such as PALB2, RAD51C, and RAD51D, may result in a similar phenotype to BRCA mutations [7].

The evolving understanding of HRR has increasing clinical importance, as an improved understanding of HRR increases potential therapeutic targets. Tumors with homologous repair deficiency (HRD) have increased sensitivity to both DNA-damaging agents and poly (ADP-ribose) polymerase (PARP) inhibitors, the topic of this review [8]. This article aims to review the current literature surrounding the use of PARP inhibitors (PARPis), their efficacy, limitations, and future perspectives.

## Indications

PARPis are currently approved to treat ovarian cancer, castration-resistant metastatic prostate cancer, metastatic pancreatic cancer, and breast cancer [9]. At present, two PARPis are approved for the treatment of breast cancer: olaparib and talazoparib. In 2018, olaparib was approved in the USA for patients with metastatic HER2-negative breast cancer with germline BRCA mutations, following the promising results of the OlympiAD trial [10••, 11]. In Europe, it is also approved for locally advanced breast cancer [12]. One year later (2019), talazoparib was approved for the same patient group following the EMBRACA trial [13••, 14]. Both trials showed improved progression-free survival (PFS) and health-related quality of life compared to chemotherapy; however, overall survival was not significantly improved [15].

Specifically, the OlympiAD trial randomized 302 patients, in a 2:1 ratio, to receive olaparib monotherapy vs. single-agent chemotherapy of physician’s choice. Olaparib monotherapy had a median PFS of 2.8 months longer and a 42% lower risk of disease progression than chemotherapy [10••]. The EMBRACA trial randomized 431 patients, in a 2:1 ratio, to receive talazoparib vs. single-agent chemotherapy of the physician’s choice. Findings were similar to the OlympiAD trial, with a 3-month increase in median PFS in the talazoparib treatment group and a 46% lower risk of disease progression compared to standard chemotherapy [13••].

In 2022, following the OlympiA trial, olaparib also gained license for high-risk early HER2-negative breast cancer with a germline BRCA mutation. This phase III trial randomized 1836 patients, in a 1:1 ratio, to receive adjuvant olaparib vs. placebo in patients with high-risk clinicopathological features, early HER2-negative breast cancer with a germline BRCA mutation, post-local treatment plus adjuvant/neoadjuvant chemotherapy [11]. At 3 years follow-up, improved invasive disease-free survival (85.9% vs. 77.1%) and distant recurrence-free survival (87.5% vs. 80.4%) were observed in the olaparib group vs. placebo [11, 16] (Table 1).

**Table 1:** Approved PARPis in breast cancer

Generic name	Trade name	Dose	Administration	Form	Monitoring	Indications	Contraindications
Olaparib [11]	Lynparza®	300 mg BD 200 mg BD in moderate renal impairment Unstudied in severe/end-stage renal impairment 100 mg BD with strong CYP3 inhibitor 150 mg BD with moderate CYP3 inhibitor	Oral with or without food	100 mg and 150 mg capsule	Full blood count (FBC) prior to starting, repeat monthly for the first year of treatment, then as required clinically	**High-risk early breast cancer**—monotherapy/in combination with anti-estrogen therapy in HER2-negative breast cancer with germline BRCA1/2 mutation post prior neoadjuvant/adjuvant chemotherapy **Metastatic/locally advanced breast cancer**—monotherapy in HER2-negative breast cancer with germline BRCA1/2 mutation*. Not licensed for locally advanced breast cancer in the USA	Nil
Talazoparib [14]	Talzenna®	1 mg OD 0.75 mg OD in moderate renal impairment 0.5 mg OD in severe renal impairment Unstudied in end-stage renal impairment 0.75 mg OD when administered with P-glycoprotein inhibitor (P-gp)	Oral with or without food	0.25 mg, 0.5 mg, 0.75 mg, 1 mg capsule	FBC prior to starting, repeated monthly/as clinically indicated	**Metastatic/locally advanced breast cancer**—monotherapy in HER2-negative breast cancer with germline BRCA1/2 mutation*	Nil

*Must receive prior taxane and anthracycline in a neoadjuvant/metastatic setting (if suitable). Hormone-positive patients must have had disease progression post/during endocrine therapy unless unsuitable for same

While not currently licensed in breast cancer, rucaparib and niraparib are the other two PARPis approved for other cancer types. Rucaparib is approved in recurrent ovarian cancer for patients with epithelial ovarian, fallopian tube, or primary peritoneal cancer, who had a complete or partial response to platinum-based chemotherapy and in metastatic castration-resistant prostate cancer with a BRCA mutation, previously treated with androgen receptor-directed therapy and a taxane-based chemotherapy [17•]. RIO (ISRCTN92154110) is an ongoing phase II window trial assessing rucaparib response in metastatic TNBC or breast cancer with a BRCA mutation. Patients receive 12–14 days of rucaparib prior to primary neoadjuvant treatment. Tumor Ki67 is assessed pre- and post-treatment to evaluate response [18]. Niraparib is approved in both advanced and recurrent ovarian cancer for patients with epithelial ovarian, fallopian tube, or primary peritoneal cancer who had a complete or partial response to platinum-based chemotherapy or have completed three or more chemotherapy regimens of any kind and have an HRD-associated tumor [19••]. The TOPACIO/KEYNOTE-162 (NCT02657889) phase I/II trial evaluated niraparib and pembrolizumab in advanced TNBC with promising results [20].

## Mechanisms of Action

**Fig. 1:**
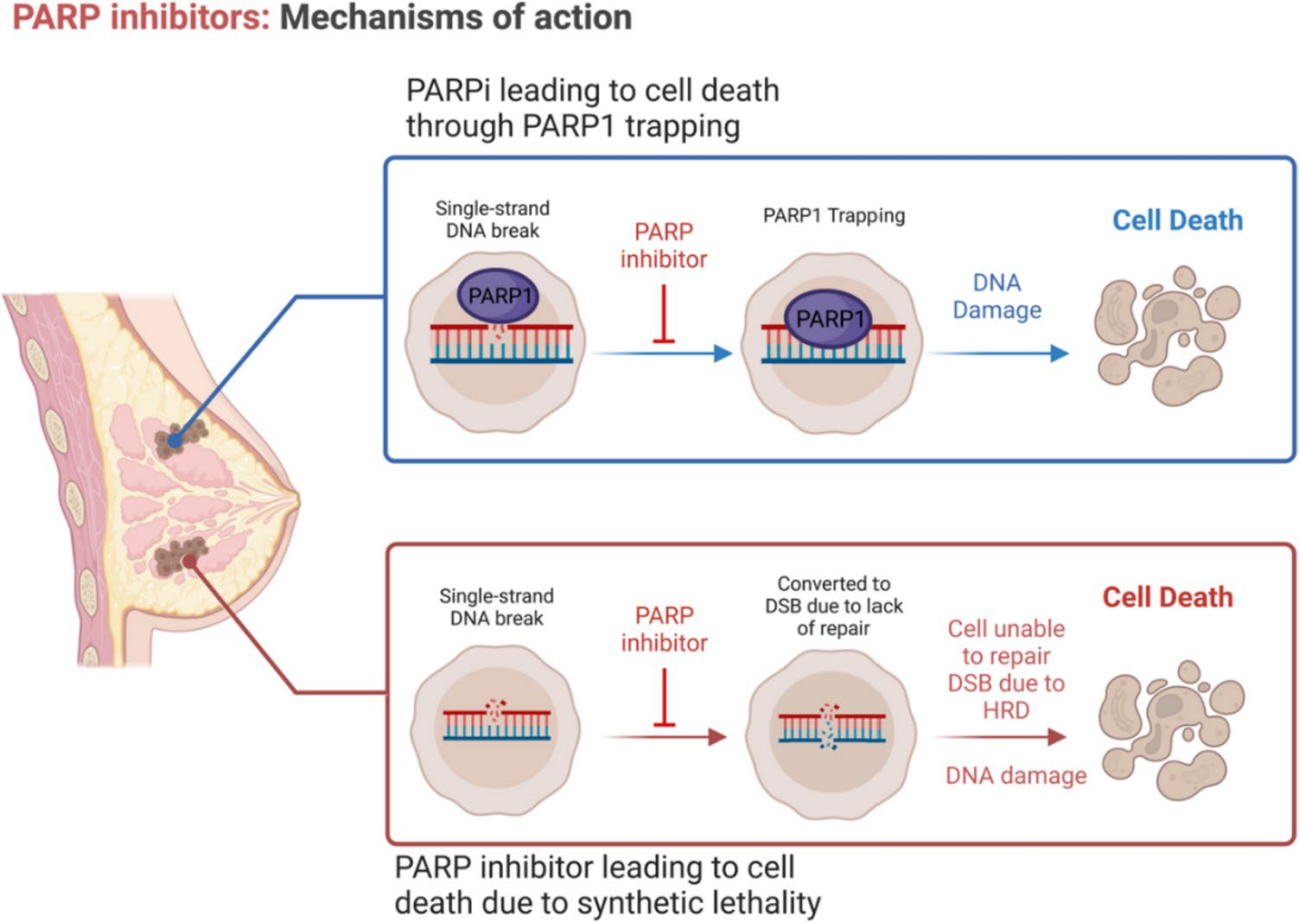
A summary of PARP inhibitor’s mechanism of action. On the top is a PARP inhibitor leading to cell death by trapping PARP1 to DNA causing DNA damage and cell death. On the bottom is the classical synthetic lethality concept, where the PARP inhibitor blocks the cell’s ability to repair single strand breaks using the base excision repair pathway, causing it to transform into a double stranded break that cannot be repaired in cells with HRD. Created with https://www.biorender.com/

When DNA damage occurs, cells have multiple repair pathways in place. These include non-homologous end joining (NHEJ), HRR, and single-strand break repair (SSBR). PARPs, specifically PARP1, PARP2, and PARP3, are critical enzymes for base excision repair (BER). They bind to damaged DNA at sites of single-stranded DNA breaks and recruit DNA repair effectors [21]. PARPis are believed to have multiple mechanisms of action. The first is through synthetic lethality, where the PARPis block BER, causing a single-stranded break to become a double-stranded break. Cells with HRD fail to repair the double-stranded break, ultimately leading to cell death. An alternative mechanism is trapping PARP1 to DNA, causing damage that HRD cells cannot repair [22]. A summary of PARPis’ mechanisms of action is displayed below in Fig. 1**.**

## Side Effects (SE) and Adverse Events (AE)

Managing the side effects and adverse events of talazoparib and olaparib provides a challenge for clinicians and requires a proactive approach to ensure both good adherence and patient safety when taking these important therapeutic agents. While all PARPis share a similar side effect profile and are tolerated similarly to chemotherapy regimens, there are some nuances between their SE/AEs and drug interaction profiles [10••, 13••, 16••, 23•]. Managing the common side effects of both olaparib and talazoparib is crucial to maintaining good adherence while preserving the patient’s quality of life. Common SEs include anemia, fatigue, nausea, and vomiting, and serious adverse events include myelodysplastic syndrome/acute myeloid leukemia (MDS/AML), pneumonitis, and venous thromboembolism (VTE) [10, 13, 16, 23].

Patients should be encouraged to be involved in their own care and counselled on how to proactively deal with SE/AEs. Strategies to combat fatigue include promoting balanced exercise, rest, and good sleep hygiene, and educating patients on the benefits of good nutrition and adequate hydration [23–26]. Other causes of fatigue, such as anemia and hypothyroidism, should be investigated. To encourage compliance, patients should be counselled that fatigue may be caused by the underlying disease process and/or chemotherapy. Nausea and vomiting may be mitigated through small, frequent meals and antiemetics [23, 24]. These symptoms may be caused by acid reflux; therefore, a proton pump inhibitor (PPI) may be of benefit. Patients should be counselled that these symptoms are worst in the first month of treatment and may improve thereafter [23, 24, 27–29].

Both talazoparib and olaparib can cause myelosuppression, including anemia, neutropenia, thrombocytopenia, lymphopenia, and myelodysplastic syndrome/acute myeloid leukemia (MDS/AML). Hematological toxicity is the most common AE necessitating dose reduction/cessation of therapy [23•]. Patients should be allowed to recover from myelosuppression adequately (≤ Grade 1) caused by prior chemotherapy before commencing PARPi treatment [11, 14]. A baseline full blood count (FBC) prior to initial treatment to assess for hematological toxicity, followed by monthly FBCs to monitor for same for the first year of treatment and then periodically thereafter [11, 14]. In mild myelosuppression, stopping medication for 1–2 days and a dose reduction should be considered [11, 14]. Dietary advice and iron supplementation may also be of benefit. If severe hematological toxicity or dependence on blood transfusion develops, it is advised to stop treatment and to investigate appropriately [11, 14]. Should blood abnormalities persist for more than 4 weeks after stopping treatment, it is advised that bone marrow and/or blood cytogenic analysis are performed [11, 14]. If AML/MDS is suspected, hematology referral is advised for an appropriate workup. If AML/MDS is confirmed, treatment should be stopped immediately [11, 12, 14, 30].

All PARPis are associated with a rare but serious risk of interstitial lung disease (ILD); however, it is most associated with olaparib administration [31]. While the occurrence has no obvious clinical pattern, it is exacerbated by having an underlying respiratory condition. Worsening respiratory symptoms or abnormal chest radiograph requires prompt investigation treatment interruption. PARPi treatment should be stopped entirely if pneumonitis is confirmed [11, 12, 31, 32]. Drug-induced liver injury (DILI) has been reported in olaparib but not talazoparib. If signs and symptoms of DILI emerge, treatment should be interrupted. If severe DILI is confirmed, treatment must be stopped [11, 12].

The drug interactions of olaparib, particularly with CYP3A inhibitors or inducers, demand careful consideration. Avoiding these interactions is preferred, but dose adjustments according to clinical guidelines are required if necessary [11, 12]. Similarly, when prescribing talazoparib, it is recommended to stop any P-glycoprotein inhibitors where possible. Where this is not feasible, talazoparib dose reduction according to guidelines is recommended [14, 30].

Certain special populations require specific counselling surrounding PARPi treatment. PARPis cause embryofoetal toxicity in laboratory studies and therefore carry significant risk if taken during pregnancy [11, 14]. Two reliable forms of contraception are strongly advisable for women of child-bearing age while on treatment and should be continued for 6 months after. Similar advice must be offered to male patients and their female partners; however, contraception needs to be continued for only 3 months after completion of treatment [11, 14]. Lactating women should be advised against breastfeeding while on treatment and for 1 month afterward due to the risk of adverse reactions in infants [11, 14]. Dose adjustment is recommended for both drugs in renal impairment; however, olaparib is unstudied in severe renal disease [11, 12, 14, 30].

Managing patients on olaparib or talazoparib involves a proactive approach, with regular monitoring and patient education paramount. Effective communication between healthcare providers and patients about symptom management and seeking timely medical advice is essential. Provision of patient information leaflets and access to support groups enable patients to be more involved in their own care. A careful balance of vigilant monitoring and personalized care plans is integral in maximizing the therapeutic benefits while minimizing the risks associated with PARPi treatment (Table 2).

**Table 2:** Side effects and adverse events. This table summarizes the common and serious side effects/adverse events associated with PARPis used in breast cancer treatment. Incidence data taken from OlympiAD [10••] (olaparib in metastatic breast cancer), OlympiA [16••] (olaparib in high-risk early breast cancer), and EMBRACA [13••] (talazoparib in metastatic breast cancer)

Side effect	Incidence	Management	Notes	References
Nausea Vomiting	OlympiAD (58%) OlympiA (56.9%) EMBRACA (48.6%) OlympiAD (29.8%) OlympiA (22.6%) EMBRACA (24.8%)	Supportive care, dietary advice, anti-emetics, PPIs, interruption/dose adjustments if severe.	Common, typically occur early in treatment, lessen over time. May be related to reflux.	[23, 24, 27–29]
Diarrhea	OlympiAD (20.5%) OlympiA (17.6%) EMBRACA (22.0%)	Hydration, dietary adjustments, anti-motility agents. Interruption and dose adjustment if severe.		[33]
Fatigue	OlympiAD (28.8%) OlympiA (40.1%) EMBRACA (50.3%)	Exercise, rest, psychosocial support, and treatment of underlying causes.	Common, usually low-grade, but persistent. May be caused by underlying disease process.	[23–26]
Anemia Neutropenia Leukopenia	OlympiAD (40.0%) OlympiA (23.5%) EMBRACA (52.8%) OlympiAD (27.3%) OlympiA (16.0%) EMBRACA (34.6%) OlympiAD (16.1%) OlympiA (15.7%) EMBRACA (17.1%)	Monitoring, treatment interruption, or cessation if severe/not resolving. Iron supplementation and blood transfusions.	Baseline full blood count (FBC), followed by monthly FBC up to 1 year and periodically thereafter. Refer to individual PARPi summary of product characteristics (SPC) for dose reduction/interruption guidelines.	[11, 12, 14, 30, 33]
Headache	OlympiAD (20.0%) OlympiA (19.8%) EMBRACA (32.5%)	Advise hydration and investigate for cause. Dose reduction if considerably impacting quality of life.		[34]
Decreased appetite	OlympiAD (16.1%) OlympiA (13.1%) EMBRACA (21.3%)	Dietary advice and nutritional supplements		[35]
MDS or AML	OlympiAD (0%) OlympiA (0.2%) EMBRACA (0%)	Monitor, hematologic consultation, and treat as per guidelines.	Risk of treatment-related myeloid neoplasms. Increased risk with previous chemotherapy.	[36, 37]
Pneumonitis	OlympiAD (no data) OlympiA (1%) EMBRACA (no data)	Hold treatment and investigated if suspected immediately. Discontinue PARPi and start corticosteroids if confirmed.	Resembles interstitial lung disease. Most cases occur within the first six months.	[11, 12, 31, 32]
VTE	OlympiAD (no data) OlympiA (no data) EMBRACA (0.3%)	Monitor high-risk patients. Anti-coagulate as per guidelines	Rare but adverse event in associated with all PARPis. Prior occurrence increases risk.	[11, 12]

## Resistance

**Fig. 2:**
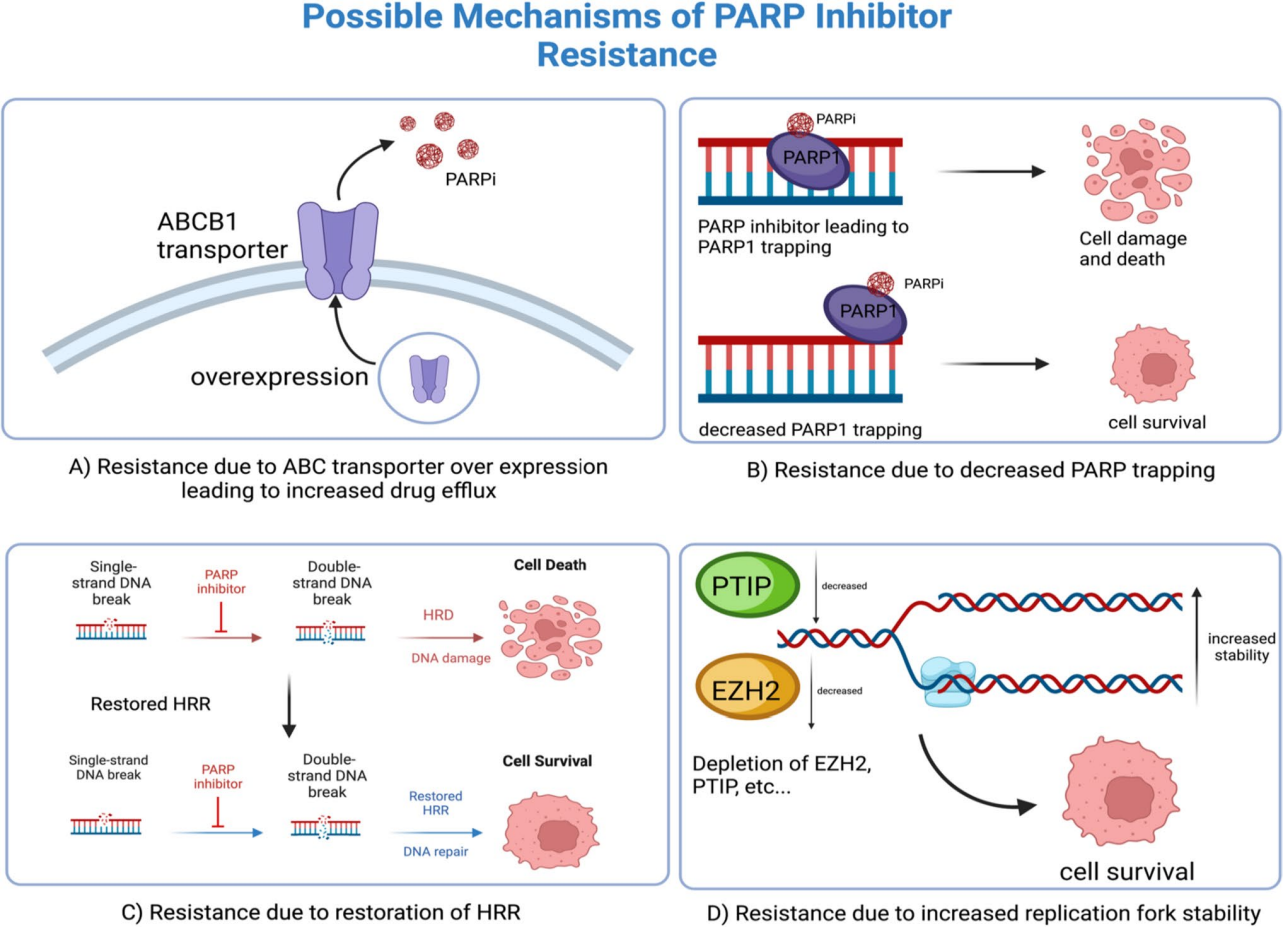
Common mechanisms behind PARP inhibitor resistance. **A** ABCB1 transporter overexpression leads to increased drug efflux. **B** PARP1 trapping is one of the mechanisms PARP inhibitors cause damage to cells; decreased PARP trapping, therefore, is a cause of resistance. **C** Restoration of HRR leads to tumor cells being able to repair the double-strand breaks caused by the PARP inhibitor. **D** Increased replication fork stability, for example, due to decrease in PTIP or EZH2, leads to increased cell survival and resistance to PARP inhibitors. Created with https://www.biorender.com/

The evolution of resistance to PARPis poses a significant problem in cancer therapeutics. Various mechanisms contribute to this resistance, as depicted in Fig. 2, such as increased drug expulsion via ABC (ATP Binding Cassette) transporters, decreased PARP1 trapping, and reactivation of HRR in HRD cells, among others. These can occur through different biological routes, including the reactivation of BRCA1/2, loss of 53BP1, and stabilization of stalled replication forks. Notably, HRD tumors, while initially more susceptible to DNA-damaging agents, may exhibit cross-resistance to PARPis and platinum-based chemotherapy, as observed in ovarian cancer studies [38].

### ABC Transporters

ABC transporters are a family of proteins that exploit ATP hydrolysis to transport various drugs across cellular membranes and hence play a significant role in multidrug resistance (MDR) in cancer by reducing intracellular drug accumulation and effectiveness. Emerging evidence suggests that ABC transporters are involved in developing resistance to PARPis in specific cancer cells. For example, one particular study demonstrated that overexpressing ABCB1 contributed to olaparib resistance in BRCA-deficient mouse mammary tumors [39]. In addition, a recent study revealed that overexpressing ABCB1 in ovarian cancer cell lines promoted resistance to niraparib [40]. These studies provide evidence that ABC transporters, such as ABCB1, can contribute to PARPi resistance in specific cancer cells by reducing the intracellular concentration of the drug. However, no clinical trials currently involve ABC inhibitors and PARPis. Therefore, further research is needed to identify strategies to overcome this resistance mechanism, such as using combination therapies and developing new or chemically modifying current PARPis that are not substrates for ABC transporters.

### Decreased PARP Trapping

Another common mechanism behind PARPi resistance is decreased PARP trapping, as PARPis’ efficacy is partly due to their ability to trap PARP proteins. Decreased PARP trapping is mainly credited to the alterations in the PARP enzymes or their interacting partners, resulting in reduced drug effectiveness. For example, one study demonstrated that the potency of PARPis in trapping PARP enzymes correlates with their cytotoxicity [41]. Moreover, the first clinical evidence of a functional link between PARP trapping and PARPi resistance was proposed by Pettitt et al. [42]. They identified a PARP-1 mutation (R591C) commonly observed in PARPi-resistant patient tumor samples, which was associated with diminished trapping of PARP-1 on DNA, resulting in PARPi resistance [42]. This finding suggests that PARP-1 mutations can decrease DNA trapping and induce PARPi resistance.

### Reactivating Homologous Repair

The reactivation of HRR in HRD cells is closely associated with resistance to PARPis, countering the therapeutic strategy these inhibitors target. This resistance often arises from reversion mutations—secondary mutations in BRCA1 or BRCA2 genes that reinstate HRR functionality—observed in patients who have developed resistance to PARPis [43]. A study on olaparib’s effectiveness in BRCA-mutated ovarian cancer revealed a higher clinical benefit in platinum-sensitive tumors (69.2%) compared to platinum-resistant (45.8%) and platinum-refractory tumors (23.1%) [44]. Furthermore, an exploratory analysis of the ARIEL2 trial demonstrated that platinum-sensitive, BRCA-mutated ovarian cancer patients treated with rucaparib experienced a median progression-free survival (PFS) of 9.4 months, in contrast to 7.2 months for those with platinum-resistant or refractory disease [45]. Conversely, the SOLO2 trial, which focused on the maintenance of olaparib in patients with BRCA-mutated, platinum-sensitive, relapsed ovarian cancer, found that disease progression post-maintenance therapy was less likely to respond to platinum-based chemotherapy in those treated with PARPis compared to those who were not [46]. This resistance is likely a consequence of shared mechanisms between PARPis and platinum-based chemotherapy, such as the reverse mutations of BRCA1/2, which restore the coding sequence of the BRCA genes, allowing for functional BRCA protein production and HRR reactivation [47]. The upregulation of HRR-associated genes like RAD51 can also play a role in overcoming the loss of BRCA function, thus contributing to resistance against PARPis. Such upregulation has been detected in BRCA-deficient cell lines and patient-derived tumor xenografts following treatment with PARPis [8]. Additionally, epigenetic modifications, including changes in DNA methylation or histone configurations, can re-establish HRR activity in HRD cells. For instance, the loss of the methyltransferase complex MLL3/4 (PTIP) in BRCA1-mutated cells can shield against DNA damage and is pivotal in developing resistance to PARPis [48]. Interestingly, the partial restoration of HRR and resistance to DNA-damaging agents have also been linked to the loss of 53BP1 in BRCA-deficient cells [49].

### Increased Replication Fork Stability

Replication fork stabilization has recently been identified as a suitable compensatory PARPi resistance mechanism without restored HRR. Recent evidence showed that the degradation of stalled replication forks is associated with PARPi sensitivity even in tumor cells without BRCA1/2 mutations [50]. In addition, the same study revealed how PARPi resistance is related to the loss of 53BP, which led to restoring RAD51 foci formation and increased replication fork stability [50]. Moreover, the loss of PTIP [48] and EZH2 [51] has contributed to PARPi resistance. Therefore, targeting restored HRR and fork protection may help combat PARPi resistance with combination therapy.

## Future Outlooks

There is much room for expanding the utility and licensing of PARPis for breast cancer patients. Three future perspectives will be discussed in this section: the use of PARPis in non-BRCA HRD, the use of PARPis alongside other agents to tackle resistance, and the use of PARPis in combination with new novel treatments.

PARPis are licensed in breast tumors with HRD with only a germline BRCA mutation status. HRR is an important DNA damage repair pathway that uses sister chromatids during the late S to G2 phase for the high-accuracy repair of double-stranded breaks. It is considered one of the only error-free double-stranded break repair mechanisms [52]. This pathway involves multiple proteins, including BRCA1, BRCA2, proteins of the MRN complex, CtIP, RAD51, ATM, H2AX, PALB2, RPA, RAD52, and proteins of the Fanconi anemia pathway [53].

When cells have HRD, they undergo double-stranded DNA break repair using the NHEJ pathway, which is highly error-prone, leading to the accumulation of mutations. The most common causes discovered thus far are loss of function mutations in BRCA1, BRCA2, RAD51C, RAD51D, PALB2, and promoter hypermethylation of BRCA1 [54].

With the evolving knowledge of HRD, the scope of use for PARPis might be expanded. For example, the PARPi rucaparib has been shown to have a clinical response in ovarian cancer patients with RAD51C mutations or methylation in phase II clinical trial Ariel 2 part 1 [55]. In addition, the phase II trial TBCRC 048 showed that PARP inhibition is an effective treatment for metastatic breast cancer patients with germline PALB2 or somatic BRCA mutations [56]. Therefore, while further studies are needed to prove the exact mechanism and gene targets, the use of PARPis in breast cancer can perhaps be expanded beyond germline BRCA mutations, increasing their utility. Various genes and pathways to enhance PARPi sensitivity are displayed in Table 3**.**

**Table 3:** List of studies that have shown PARPi sensitivity beyond germline BRCA mutations

Study name	Gene mutation	PARPi	Cancer type
Ariel 2 part 1 [55]	RAD51c	Rucaparib	Ovarian cancer
TBCRC 048 [56]	Germline PALB2 and somatic BRCA	Olaparib	Breast cancer

The improved understanding of the mechanism of PARPi resistance has enabled several studies to evaluate methods for overcoming it. As previously mentioned, the increased expression of ABC causes PARPi resistance by increasing drug efflux. A recent study has found that the co-administration of the P-glycoprotein inhibitor ondansetron can reverse this [39]. Another found that the ABCB1 inhibitors verapamil and elacridar reverse resistance in ovarian cancer cell lines overexpressing ABCB1 [40]. Another potential strategy is the combination of a PARPi with anti-CSF-1R, as it was shown to overcome resistance in BRCA1-deficient triple-negative breast cancer [57]. Therefore, while further investigation and clinical trials are necessary, the ability to reduce resistance to PARPis is both promising and evident.

Currently, PARPis are licensed in HER2-negative breast cancer only; however, aside from its role in DNA repair, PARP1 is known to influence tumor proliferation and HER2 resistance by co-activation of NF-κB [58]. NCT03368729 is a phase Ib/II study of niraparib and trastuzumab in HER2-positive, BRCA wildtype breast cancer [59]. This trial resulted from prior research by the same group which identified that HER2-positive tumors overexpress PARP1 and that PARPis induced tumor apoptosis in HER2-positive animal and cell line models irrespective of mutational status [60]. The trial is currently recruiting and includes patients who have had disease progression after receiving at least one anti-HER2 therapy in the metastatic setting [59].

Lastly, many other trials are investigating the efficacy of PARPis in combination with other drugs (summarized in Table 4). For example, the BROCADE3 trial tested the combination of veliparib with carboplatin and paclitaxel in patients with BRCA-mutated advanced breast cancer and found a significant improvement in progression-free survival [61]. Moreover, this is not limited to chemotherapeutic agents; immune checkpoint inhibitors (ICIs) have already demonstrated efficacy when combined with chemotherapy in triple-negative breast cancer in the KEYNOTE-522 clinical trial [62]. Immune checkpoint inhibition, such as programmed death (PD)-1 and PD-ligand 1 (PD-L1) pathway blockade, has led to significant clinical advances in the treatment of solid tumors [63]. However, one of the major challenges of this approach is the limited single-agent activity in many cancers, leaving the opportunity to test combinations [63]. Combining ICIs and PARPis is an area of potential research to exploit their different mechanisms of action and enhance the overall effectiveness of the therapy. Ongoing clinical trials are investigating the safety, efficacy, and optimal dosing strategies for combining these two classes of drugs to maximize their therapeutic potential. The TOPACIO/KEYNOTE-162 trial demonstrated that combining the PARPi niraparib and the ICI pembrolizumab effectively treated patients with advanced TNBC, suggesting an enhanced immune response against cancer cells with DNA damage [64]. Moreover, an early-phase clinical trial revealed that combining the PARPi olaparib and the PD-L1 inhibitor durvalumab was considered tolerable and demonstrated clinical activity in patients with advanced women’s cancers, signifying potential synergy between PARPis and ICIs in overcoming PARPi resistance [65]. One previous study showed that PARPis treatment in BRCA1-deficient tumors could stimulate an immune response against cancer cells by activating the STING pathway. Therefore, ICIs can enhance this immune activation, leading to more effective anti-tumor responses [66].

**Table 4:** Examples of clinical trials that tested a combination of PARPi with another drug

Study name	PARPi	Combination drug	Cancer type	Outcome
NCT03368729 [59]	Niraparib	Trastuzumab	Metastatic HER2-positive breast cancer	Currently recruiting
BROCADE3 [61]	Veliparib	Carboplatin and paclitaxel	BRCA-mutated advanced breast cancer	“The addition of veliparib to a highly active platinum doublet, with continuation as monotherapy if the doublet were discontinued, resulted in significant and durable improvement in progression-free survival.”
MEDIOLA [67]	Olaparib	Durvalumab	Breast cancer with germline BRCA mutation	“Combination showed promising antitumor activity.”
TOPACIO [20]	Niraparib	Pembrolizumab	Advanced triple-negative breast cancer	“Combination niraparib plus pembrolizumab provides promising antitumor activity in patients with advanced or metastatic TNBC.”
PAOLA-1 [68]	Olaparib	Bevacizumab	Maintenance in ovarian cancer	“Administering maintenance olaparib in addition to bevacizumab to patients with newly diagnosed advanced ovarian cancer who were receiving standard treatment including bevacizumab resulted in a significant progression-free survival benefit, with a substantial benefit in patients with HRD-positive tumors.”

## Conclusion

Overall, PARPis are an effective and well-tolerated treatment option for certain breast cancers. The improved understanding of HRD, increased ability to target resistance, and combination of PARPis with other novel agents will continue to expand their use. In addition, the results of ongoing trials may allow PARPis to become an even more pivotal therapeutic option in treating breast cancer [10, 13, 16, 20, 39, 40, 55–57, 61, 64–68].
